# A New Marker for Determining Cardiovascular Risk: Salusin Alpha

**DOI:** 10.7759/cureus.30340

**Published:** 2022-10-16

**Authors:** Emre Yılmaz, Devrim Kurt, Ertan Aydın, Sencer Çamcı, Aslı Vural

**Affiliations:** 1 Cardiology, Giresun University Faculty of Medicine, Giresun, TUR

**Keywords:** pce, score2, ischemic heart disease, salusin alpha, cardiovascular risk

## Abstract

Background and objective

Prevention of atherosclerotic cardiovascular diseases (ASCVD) is possible with early recognition of individuals at risk. Salusin alpha is an endogenous bioactive peptide with anti-atherogenic properties. We aimed to reveal the relationship between salusin alpha levels and cardiovascular (CV) risk using the Systematic COronary Risk Estimation 2 (SCORE2) and Pooled Cohort Equation (PCE) algorithms.

Methods

A total of 137 asymptomatic outpatients were included in the study. The participants were divided into four quartile groups according to the distribution of salusin alpha levels: Q1 (n = 34), Q2 (n = 35), Q3 (n = 34), and Q4 (n = 34). The relationships of salusin alpha with cardiovascular risk scores and groups were investigated.

Results

The means of SCORE2 and PCE risk scores (11.24 ± 1.24 and 13.30 ± 1.71, respectively; p < 0.001 for both), baseline scores in the presence of SCORE2 and PCE optimal risk factors (4.82 ± 0.71 (p = 0.034); 4.20 ± 0.77 (p = 0.010)), and means of Δ SCORE2 and Δ PCE risk score (6.41 ± 0.67 and 9.10 ± 1.10, respectively; p < 0.001 for both) were significantly higher in the Q1 group. The SCORE2 “very high” cardiovascular (CV) risk group was significantly represented in the Q1 group (n = 17 (50%)), while the “low-moderate” risk group was more heavily represented in the Q4 group (n = 15 (44.1%)) (p < 0.001). The PCE “high” CV risk group was significantly represented in the Q1 group (n = 9 (26.5%)), while the “low” risk group was more intensely represented in the Q4 group (n = 20 (58.8%)) (p < 0.001). Salusin alpha had a significantly negative moderate correlation with SCORE2 and PCE risk scores. As a result of receiver operating characteristic (ROC) analysis, it was found that salusin alpha had significant diagnostic power in the prediction of CV risk groups determined by SCORE2 and PCE risk scores. Salusin alpha was observed to be a risk-reducing factor in SCORE2 and PCE CV risk groups (odds ratio (OR) (95% confidence interval (CI)): 0.989 (0.982-0.997) (p = 0.002) and OR (95% CI): 0.988 (0.978-0.998) (p = 0.009) respectively).

Conclusion

Salusin alpha has a negative correlation with SCORE2 and PCE risk scores. It has significant diagnostic power in the prediction of CV risk groups and is an independent variable that has a risk-reducing effect in the distribution of risk groups.

## Introduction

Cardiovascular diseases (CVD), especially ischemic heart disease, are the leading causes of death worldwide [1]. Ischemia often develops as a result of occlusive atherosclerotic vascular disease. Although the incidence of atherosclerotic cardiovascular disease (ASCVD) has decreased in many European countries in recent years, it is still an important cause of mortality and morbidity [2]. The prevention of CVD before it causes serious symptoms and complications can only be possible with early recognition of individuals at risk. Various algorithms have been described so far to determine the risk of ASCVD. Among them, the algorithm known as Systematic COronary Risk Estimation 2 (SCORE2) was defined and put into use by the European Society of Cardiology (ESC) in 2021 for the primary prevention of CVD in all European countries. The SCORE2 algorithm predicts fatal and nonfatal (myocardial infarction and stroke) cardiovascular (CV) events that may develop within the next 10 years in apparently healthy individuals over 40 years of age without serious comorbidities and ASCVD. In this algorithm, cardiovascular risk is calculated using data such as age, gender, smoking habit, systolic blood pressure, and non-high-density lipoprotein (non-HDL) cholesterol level. The routine use of any biochemical parameter other than non-HDL cholesterol in the algorithm is not recommended by the ESC [2]. The Pooled Cohort Equation (PCE) was created by the American College of Cardiology and the American Heart Association in 2013 using various patient cohorts. This risk model was developed in the United States with large white and Afro-American communities. It is calculated by variables of age, gender, ethnicity, systolic and diastolic blood pressures, total cholesterol, high-density lipoprotein (HDL) and low-density lipoprotein (LDL) cholesterol, smoking, history of diabetes, and hypertension treatment [3,4].

Salusins are endogenous bioactive peptides that are synthesized in tissues such as human vascular endothelial cells, kidneys, and brains and have two forms (alpha and beta), first described by Shichiri et al. [5] in 2003. The first clinical study showing the relationship between these peptides and atherosclerosis was conducted by Watanabe et al. [6] and included patients with angiographically proven coronary artery disease (CAD) and a control group. In this study, salusin alpha level was found to be significantly lower in the group with CAD. It was shown that salusin alpha has anti-atherogenic properties, while salusin beta has pro-atherogenic effects. This opposite effect was also observed in an animal experiment by Nagashima et al. [7], in which salusins were administered exogenously. Evidence suggests a negative association between atherosclerosis and plasma salusin alpha level. In the case of atherosclerosis, while the plasma salusin alpha level decreases, on the contrary, when its plasma levels increase, it is understood that atherosclerosis is suppressed.

In this study, we tried to evaluate the relationship between cardiovascular risk level and plasma salusin alpha level in asymptomatic and apparently healthy individuals who were not previously diagnosed with ASCVD by clinical or imaging methods and did not have diabetes and serious comorbid diseases.

## Materials and methods

Study design

Asymptomatic participants over the age of 40 who applied to the cardiology outpatient clinic between February 2020 and June 2021 were included in the study. The exclusion criteria were CVD (coronary artery disease, stroke, history of transient ischemic attack, and peripheral artery disease), diabetes mellitus, familial hypercholesterolemia, estimated glomerular filtration rate (eGFR) < 60 mL/minute/1.73 m², severe liver disease, malignancy, heart failure, severe heart valve disease, and active infection. A total of 137 participants were included in the study. Age, gender, smoking habit, laboratory results, and echocardiography measurements were obtained at the time of the first application. The height and weight of the participants were measured, and body mass index (BMI) was calculated by dividing the body weight by the square of the height (kg/m²). In all participants, arterial blood pressure was measured from the upper arm in the outpatient clinic with a standard cuff size aneroid sphygmomanometer (ERKA Perfect Aneroid, ERKA, Bad Tölz, Germany). The risk of ASCVD in all participants was determined as recommended by guidelines on cardiovascular disease prevention published by the ESC in 2021 [2]. SCORE2 scores corresponding to age, gender, smoking, systolic blood pressure, and non-HDL cholesterol level parameters were calculated. For this, the SCORE2 risk table was used for participants aged 40-69, and the SCORE2-Older Persons (SCORE2-OP) risk table for participants aged over 70, as recommended by the guideline. In addition, PCE risk score calculations were made from the study participants. Optimal risk scores were calculated by assuming that participants’ other risk factors were optimal based on non-modifiable risk factors. These scores were the optimal scores of the relevant age and gender without additional risk factors and were named “SCORE2 optimal” and “PCE optimal.” Deviation from optimal risk scores due to non-modifiable risk factors such as age, gender, and ethnicity was calculated using the following formulas: Δ SCORE2 = SCORE2 - “SCORE2 optimal” and Δ PCE = PCE - “PCE optimal.”

The participants were divided into four groups in 25% quartiles according to the distribution of salusin alpha levels. The group with the lowest salusin alpha level was expressed with Q1 and the highest group with Q4. Echocardiographic evaluations of the participants were performed in the left lateral position using the standard approach. Left ventricular end-diastole (LVED), left ventricular end-systole (LVES), and left atrium (LA) diameters were measured from the parasternal long axis window using the m-mode method. The ejection fraction (EF) was determined using the modified Simpson method.

This study was approved by the local ethics committee (Clinical Research Ethics Committee of Giresun University) (approval date - number: 04/09/2018 - 01/06); all patients were informed about the goals of the study, and informed consent was obtained.

Laboratory procedures

Venous blood samples were taken from all patients after 12 hours of fasting, and serum creatinine, glycated hemoglobin (HbA1c), aspartate aminotransferase (AST), alanine aminotransferase (ALT), total cholesterol (TC), triglyceride (TG), HDL, LDL, white blood cell (WBC), hemoglobin (HGB), and platelet (PLT) levels were determined. Non-HDL level was found by subtracting HDL from TC. eGFR was calculated using the Modified Diet in Renal Disease (MDRD) formula [8]. Plasma salusin alpha levels were analyzed using an enzyme-linked immunosorbent assay reagent kit (Abbkine, Inc., Wuhan, China). The detection range of the kit was 20-320 pg/mL, and the plasma samples were studied at a 1:5 dilution ratio. Plasma salusin alpha levels were expressed as pg/mL.

Statistical analysis

Analysis was performed using the Statistical Package for the Social Sciences version 21 software (IBM SPSS Statistics, Armonk, NY, USA). The normal distribution of continuous variables was evaluated using the Kolmogorov-Smirnov test. Continuous variables with normal distribution were given as mean ± standard deviation, non-normally distributed variables were given as median (25th-75th quartiles), and categorical variables were given as percent (%). The chi-square test was used for categorical variables. One-way analysis of variance (ANOVA) or the Kruskal-Wallis test was used for statistical analysis of clinical data between groups. The Pearson correlation coefficient was used for correlations, and Tukey’s test was used for post hoc analyses. The correlation between salusin alpha with SCORE2 and PCE risk scores was evaluated with a scatterplot. The optimal cutoff value of salusin alpha for predicting CV risk groups was evaluated using receiver operating characteristic (ROC) analysis. The factors affecting the distribution of CV risk groups were investigated using logistic regression analysis. Variables with a p-value of <0.05 in univariate analyzes were included in the multivariate analysis model. A p-value of <0.05 was considered statistically significant.

## Results

The participants included in our study were evaluated by dividing them into four quarter groups of 25% according to the distribution of salusin alpha levels. The number of participants and salusin alpha levels of the groups were as follows: 34 participants in the Q1 group, with a mean salusin alpha level of 274.52 ± 16.44 pg/mL; 35 participants in the Q2 group, with a mean salusin alpha level of 319.73 ± 12.21 pg/mL; 34 participants in the Q3 group, with a mean salusin alpha level of 371.96 ± 16.88 pg/mL; and 34 participants in the Q4 group, with a mean salusin alpha level of 498.38 ± 64.31 pg/mL (p < 0.001). The groups were similar in terms of age, gender, and BMI. Smoking (n = 18 (52.9%)), SCORE2 mean risk score (11.24 ± 1.24), baseline score in the presence of SCORE2 optimal risk factors (4.82 ± 0.71), and mean Δ SCORE2 (6.41 ± 0.67) were significantly higher in the Q1 group (p = 0.004, p < 0.001, p = 0.034, and p < 0.001, respectively). In the distribution of SCORE2 CV risk groups, it was observed that the “very high” risk group was significantly represented in the Q1 group (n = 17 (50%)), and the “low-moderate” risk group was more intensely represented in the Q4 group (n = 15 (44.1%)) (p < 0.001). The mean PCE risk score (13.30 ± 1.71), the baseline score in the presence of PCE optimal risk factors (4.20 ± 0.77), and the mean Δ PCE (9.10 ± 1.10) were significantly higher in the Q1 group (p < 0.001, p = 0.010, and p < 0.001, respectively). In the distribution of PCE CV risk groups, it was observed that the “high” risk group was significantly represented in the Q1 group (n = 9 (26.5%)) and the “low” risk group was more intensely represented in the Q4 group (n = 20 (58.8%)) (p < 0.001). Lipid profiles were generally similar between groups. However, the numerical difference between the Q1 and Q4 groups was found to be significant in total cholesterol (Q1 and Q2 versus Q4), triglyceride, LDL (Q1 and Q2 versus Q4), and HDL and non-HDL cholesterol levels (Q1 and Q2 versus Q4). There was no significant difference between the groups in blood pressure, echocardiography, and other laboratory measurements. Details of demographic characteristics, echocardiography, and laboratory measurements are presented in Table 1.

**Table 1:** Demographic characteristics, echocardiography, and laboratory data of salusin alpha quartile groups. Normally distributed values are presented as mean ± standard deviation, non-normally distributed values as median (25th-75th quartiles), and categorical values as number (percentages). HT, hypertension; SCORE2, Systematic COronary Risk Evaluation 2; CV, cardiovascular; PCE, Pooled Cohort Equation; BMI, body mass index; SBP, systolic blood pressure; DBP, diastolic blood pressure; HbA1c, glycated hemoglobin; eGFR, estimated glomerular filtration rate; ALT, alanine aminotransferase; AST, aspartate aminotransferase; TC, total cholesterol; TG, triglycerides; LDL, low-density lipoprotein cholesterol; HDL, high-density lipoprotein cholesterol; WBC, white blood cell; HGB, hemoglobin; PLT, platelet; LVED, left ventricular end-diastole; LVES, left ventricular end-systole; LA, left atrium; EF, ejection fraction

Parameters	Q1 (≤298.08) (n = 34)	Q2 (298.08-338.72) (n = 35)	Q3 (338.72-402.37) (n = 34)	Q4 (>402.37) (n = 34)	p-value
Age (year)	56.29 ± 10.47	53.97 ± 8.27	54.47 ± 6.72	52.41 ± 8.01	0.235
Sex (male), n (%)	20 (58.8%)	17 (48.6%)	16 (47.1%)	10 (29.4%)	0.106
Current smoker, n (%)	18 (52.9%)	11 (31.4%)	6 (17.6%)	6 (17.6%)	0.004
HT, n (%)	22 (64.7%)	21 (60%)	26 (76.5%)	17 (50%)	0.153
SCORE2	11.24 ± 1.24	7.26 ± 0.78	7.06 ± 0.73	5.15 ± 0.57	<0.001
SCORE2 optimal	4.82 ± 0.71	3.49 ± 0.38	3.32 ± 0.35	2.97 ± 0.34	0.034
Δ SCORE2	6.41 ± 0.67	3.77 ± 0.55	3.74 ± 0.52	2.18 ± 0.39	<0.001
SCORE2 CV risk groups					<0.001
Low-moderate, n (%)	1 (2.9%)	11 (31.4%)	9 (26.5%)	15 (44.1%)	
High, n (%)	16 (47.1%)	11 (31.4%)	16 (47.1%)	17 (50%)	
Very high, n (%)	17 (50%)	13 (37.1%)	9 (26.5%)	2 (5.9%)	
PCE	13.30 ± 1.71	8.13 ± 0.99	8.24 ± 1.10	4.91 ± 0.63	<0.001
PCE optimal	4.20 ± 0.77	2.46 ± 0.28	2.86 ± 0.42	2.01 ± 0.29	0.010
Δ PCE	9.10 ± 1.10	5.66 ± 0.82	5.37 ± 0.83	2.89 ± 0.48	<0.001
PCE CV risk groups					<0.001
Low, n (%)	6 (17.6%)	16 (45.7%)	13 (38.2%)	20 (58.8%)	
Borderline, n (%)	10 (29.4%)	3 (8.6%)	3 (8.8%)	9 (26.5%)	
Intermediate, n (%)	9 (26.5%)	16 (45.7%)	16 (47.1%)	5 (14.7%)	
High, n (%)	9 (26.5%)	0	2 (5.9%)	0	
Salusin alpha (pg/mL)	274.52 ± 16.44	319.73 ± 12.21	371.96 ± 16.88	498.38 ± 64.31	<0.001
BMI (kg/m^2^)	28.61 ± 3.69	28.36 ± 4.66	29.10 ± 3.90	28.46 ± 4.22	0.878
SBP (mmHg)	143.74 ± 17.15	140.09 ± 18.35	147.38 ± 16.92	139.09 ± 16.93	0.202
DBP (mmHg)	84.35 ± 12.55	85.43 ± 12.84	86.35 ± 11.89	82.41 ± 12.23	0.554
HbA1c (%)	5.47 ± 0.47	5.59 ± 0.34	5.63 ± 0.38	5.49 ± 0.42	0.626
Creatine (mg/dL)	0.81 ± 0.15	0.79 ± 0.13	0.78 ± 0.19	0.74 ± 0.14	0.254
eGFR(mL/minute/1.73 m^2^)	93.31 ± 15.03	94.76 ± 13.05	93.98 ± 14.35	96.20 ± 12.97	0.826
ALT (IU/L)	22.68 (17.66-27.70)	21.69 (17.84-25.53)	23.32 (16.65-30)	22.53 (18.43-26.62)	0.992
AST (IU/L)	19.91 ± 5.93	21.63 ± 6.73	21.88 ± 12.43	21.26 ± 6.79	0.767
TC (mg/dL)	207.03 ± 32.72	207.66 ± 33.28	201.71 ± 49.14	190.24 ± 36.86	0.006
TG (mg/dL)	171.88 (131.93-225.81)	168.86 (128.21-214.52)	164.35 (121.09-219.43)	161.44 (123.23-215.12)	0.002
LDL (mg/dL)	123.18 ± 30.36	123.80 ± 28.70	117.76 ± 40.60	114.97 ± 34.95	0.004
HDL (mg/dL)	44.76 ± 11.64	46.49 ± 9.87	48.12 ± 12.16	50.76 ± 15	0.076
Non-HDL (mg/dL)	162.26 ± 32.36	161.17 ± 32.07	152.68 ± 46.12	139.47 ± 35.52	0.024
WBC (10^3^/mm^3^)	7.27 (6.34-8.20)	7.05 (6.31-7.79)	6.78 (6.27-7.28)	6.82 (6.15-7.50)	0.767
HGB (g/dL)	14.48 ± 1.61	14.44 ± 1.47	13.98 ± 1.81	14.05 ± 1.46	0.566
PLT (10^3^/mm^3^)	236.02 ± 47.06	241.73 ± 60.39	252.08 ± 61.06	247.35 ± 58.83	0.654
LVED diameter (mm)	47.38 ± 4.39	47.34 ± 4.02	47.12 ± 4.21	45.21 ± 3.16	0.114
LVES diameter (mm)	27.71 ± 3.60	27.54 ± 3.92	27.41 ± 4.25	27.06 ± 3.82	0.903
LA diameter (mm)	34.74 ± 5.16	35.06 ± 4.54	33.09 ± 5.01	33.82 ± 5.34	0.366
EF (%)	64.71 ± 1.19	64.71 ± 1.17	64.71 ± 1.19	64.85 ± 0.85	0.948

Salusin alpha was found to have a significant negative correlation with age (r: -0.217; p = 0.011), smoking (r: -0272; p = 0.001), and non-HDL cholesterol (r: -0.176; p = 0.040). It was found that SCORE2 and PCE risk scores were strongly correlated (r: 0.917; p < 0.001) and had a similarly significant correlation with optimal baseline score measurements, Δ measurements, and risk groups. Salusin alpha had a moderately significant negative correlation with SCORE2 and PCE risk scores. It was found that this relationship was similarly significant in optimal baseline measurements, Δ measurements, and risk groups. Correlation analysis results are presented in Table 2.

**Table 2:** Correlation of salusin alpha, and SCORE2 and PCE risk scores with other variables. SCORE2, Systematic COronary Risk Evaluation 2; PCE, Pooled Cohort Equation; HT, hypertension; CV, cardiovascular; BMI, body mass index; SBP, systolic blood pressure; DBP, diastolic blood pressure; HbA1c, glycated hemoglobin; TC, total cholesterol; TG, triglycerides; LDL, low-density lipoprotein cholesterol; HDL, high-density lipoprotein cholesterol

Parameters	Salusin alpha		SCORE2		PCE	
	r	p-value	r	p-value	r	p-value
Age (year)	-0.217	0.011	0.770	<0001	0.638	<0.001
Sex (male), n (%)	0.163	0.057	-0.077	0.373	-0.264	0.002
Current smoker, n (%)	-0.272	0.001	0.289	0.001	0.328	<0.001
HT, n (%)	-0.138	0.107	0.342	<0.001	0.375	<0.001
SCORE2	-0.395	<0001	-	-	0.917	<0.001
SCORE2 optimal	-0.245	0004	0.832	<0.001	0.720	<0.001
Δ SCORE2	-0.420	<0.001	0.894	<0.001	0.854	<0.001
SCORE2 CV risk groups	-0.441	<0.001	0.826	<0.001	0.791	<0.001
PCE	-0.374	<0.001	0.917	<0.001	-	-
PCE optimal	-0.237	0.005	0.804	<0.001	0.811	<0.001
Δ PCE	-0.391	<0.001	0.837	<0.001	0.948	<0.001
PCE CV risk groups	-0.363	<0.001	0.844	<0.001	0.878	<0.001
BMI (kg/m^2^)	0.009	0.915	0.088	0.306	0.072	0.401
SBP (mmHg)	-0.095	0.269	0.351	<0.001	0.328	<0.001
DBP (mmHg)	-0.075	0.386	0.016	0.856	0.044	0.606
HbA1c (%)	-0.057	0.507	0.049	0.569	0.042	0.623
Creatine (mg/dL)	-0.100	0.247	0.217	0.011	0.355	<0.001
TC (mg/dL)	-0.137	0.111	0.127	0.138	0.098	0.255
TG (mg/dL)	-0.142	0.097	0.031	0.715	0.129	0.134
LDL (mg/dL)	-0.067	0.435	0.116	0.177	0.097	0.261
HDL (mg/dL)	0.119	0.165	-0.008	0.925	-0.207	0.015
Non-HDL (mg/dL)	-0.176	0.040	0.136	0.112	0.170	0.047

**Figure 1:**
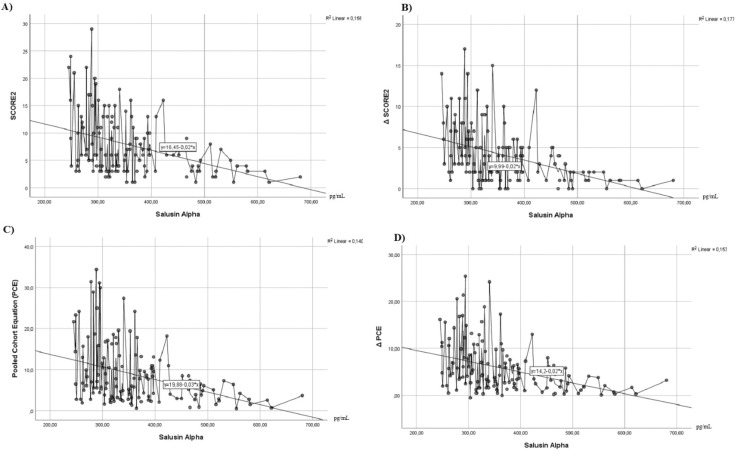
Scatterplot of the relationship between salusin alpha and SCORE2 and PCE risk scores. (A) SCORE2-salusin alpha relationship, (B) Δ SCORE2-salusin alpha relationship, (C) PCE-salusin alpha relationship, and (D) Δ PCE-salusin alpha relationship. SCORE2, Systematic COronary Risk Evaluation 2; PCE, Pooled Cohort Equation

Δ SCORE2 and Δ PCE scores obtained by the difference of the risk scores obtained with the participants’ current risk factors (SCORE2 and PCE) and the risk scores obtained by assuming other risk factors based on non-modifiable risk factors at optimal limits (SCORE2 optimal and PCE optimal) were compared with salusin alpha. It was observed that there was a higher level of negative correlation. Relevant correlation relationships are presented in Figure 1 with a scatterplot.

As a result of ROC analysis, it was found that salusin alpha had significant diagnostic power in the prediction of CV risk groups determined by SCORE2 and PCE risk scores. The strongest diagnostic power was found to be predictive of PCE high CV risk group with a cutoff value of <296.41 with 81% sensitivity and 81% specificity (area under the curve (AUC) (95% confidence interval (CI)): 0.822 (0.711-0.933); p < 0.001). ROC analysis details are presented in Table 3.

**Table 3:** Diagnostic importance of salusin alpha in the prediction of risk groups obtained by risk scores (ROC analysis details). ROC, receiver operating characteristic; AUC, area under the curve; CI, confidence interval; PCE, Pooled Cohort Equation; CV, cardiovascular; SCORE2, Systematic COronary Risk Evaluation 2

	AUC	95% CI	Sensitivity	Specificity	Cutoff	p-value
PCE high CV risk	0.822	0.711-0.933	81%	81%	<296.41	<0.001
PCE low CV risk	0.667	0.574-0.759	85%	45%	>313.20	0.001
SCORE2 very high risk	0.732	0.646-0.817	70%	65%	<332.79	<0.001
SCORE2 low-moderate risk	0.719	0.624-0.813	91%	41%	>313.20	<0.001

According to the results of the multivariate logistic regression analysis, salusin alpha, systolic blood pressure, smoking, gender, and age were determined as factors affecting SCORE2 CV risk group distribution. Salusin alpha was observed to be a risk-reducing factor in the SCORE2 CV risk grouping (odds ratio (OR) (95% CI): 0.989 (0.982-0.997); p = 0.002). Logistic regression analysis details for factors affecting the SCORE2 CV risk group distribution are presented in Table 4.

**Table 4:** Logistic regression analysis results of factors affecting CV risk groups determined by SCORE2. CV, cardiovascular; SCORE2, Systematic COronary Risk Evaluation 2; OR, odds ratio; CI, confidence interval; SBP, systolic blood pressure; DBP, diastolic blood pressure; TC, total cholesterol; TG, triglycerides; HDL, high-density lipoprotein cholesterol; LDL, low-density lipoprotein cholesterol; BMI, body mass index

	SCORE2: low-moderate versus high-very high			
	Univariate		Multivariate	
	OR (95% CI)	p-value	OR (95% CI)	p-value
Salusin alpha	0.991 (0.986-0.995)	<0.001	0.989 (0.982-0.997)	0.002
SBP	1.060 (1.031-1.090)	<0.001	1.145 (1.072-1.224)	<0.001
DBP	1.034 (1.001-1.068)	0.083		
TC	1.004 (0.994-1.014)	0.488		
HDL	0.987 (0.957-1.017)	0.378		
LDL	1.004 (0.992-1.015)	0.532		
Non-HDL	1.006 (0.996-1.016)	0.272		
Current smoke	10.69 (2.431-47.03)	0.002	12.90 (2.169-76.83)	0.001
Gender	0.415 (0.185-0.931)	0.033	0.026 (0.003-0.214)	0.001
Age	1.126 (1.063-1.193)	<0.001	1.412 (1.205-1.655)	<0.001
BMI	1.006 (0.917-1.105)	0.896		

According to the results of the multivariate logistic regression analysis, salusin alpha, systolic blood pressure, HDL cholesterol level, smoking, gender, and age were determined as factors affecting PCE CV risk group distribution. Salusin alpha was observed to be a risk-reducing factor in PCE CV risk grouping (OR (95% CI): 0.988 (0.978-0.998); p = 0.009). Logistic regression analysis details for the factors affecting the PCE CV risk group distribution are presented in Table 5.

**Table 5:** Logistic regression analysis results of factors affecting CV risk groups determined by PCE. CV, cardiovascular; PCE, Pooled Cohort Equation; OR, odds ratio; CI, confidence interval; SBP, systolic blood pressure; DBP, diastolic blood pressure; TC, total cholesterol; TG, triglycerides; HDL, high-density lipoprotein cholesterol; LDL, low-density lipoprotein cholesterol; BMI, body mass index

	PCE: low-borderline versus intermediate-high			
	Univariate		Multivariate	
	OR (95% CI)	p-value	OR (95% CI)	p-value
Salusin alpha	0.991 (0.987-0.996)	0.001	0.988 (0.978-0.998)	0.009
SBP	1.049 (1.025-1.074)	<0.001	1.172 (1.084-1.267)	<0.001
DBP	1.015 (0.987-1.043)	0.302		
TC	1.003 (0.994-1.012)	0.496		
HDL	0.971 (0.942-0.998)	0.033	0.864 (0.774-0.964)	0.009
LDL	1.002 (0.992-1.013)	0.638		
Non-HDL	1.006 (0.997-1.015)	0.170		
Current smoke	3.634 (1.689-7.819)	0.001	7. 170 (3.248-15.82)	<0.001
Gender	0.436 (0.218-0.873)	0.019	0.009 (0.001-0.107)	<0.001
Age	1.206 (1.129-1.289)	<0.001	1.761 (1.391-2.230)	<0.001
BMI	1.034 (0.951-1.123)	0.432		

## Discussion

Our aim in our study was to determine alternative methods and variables to increase the effectiveness and diagnostic power of conventional CV risk classification systems in the healthy population. The results we have obtained can bring a different perspective to the subject and prepare the ground for other studies that can be applied in the future. In our study, we found a significant negative correlation between plasma salusin alpha level and SCORE2 and PCE scores. Salusin alpha has significant diagnostic power in the prediction of CV risk groups. It is an independent variable that has a risk-reducing effect in the distribution of CV risk groups. Salusin alpha is associated not only with CV risk scores but also with participants’ scores for deviation from baseline risk status (Δ SCORE2 and Δ PCE) based on age, sex, and ethnicity. This negative correlation has a stronger correlation than standard scores.

There are many studies in the literature showing that salusins ​​are associated with atherosclerosis. In a study involving patients who underwent coronary angiography for chronic coronary syndrome, plasma salusin alpha level was found to be negatively correlated with the presence and severity of CAD. In this study, the authors emphasized that salusin alpha may be a potential biomarker in determining the risk of future CAD [9]. In two other studies including patients with ST-elevation myocardial infarction [10] and patients with carotid atherosclerosis [11], it was shown that plasma salusin alpha levels decreased in these patient populations. In animal experiments, there are studies showing that atherosclerosis is suppressed in animals treated with exogenous salusin alpha infusion [7,12,13]. These studies showing the relationship of salusins ​​with atherosclerosis include patient groups with obvious clinical findings related to atherosclerosis. There are insufficient data to evaluate the role of salusin alpha in CV risk determination in healthy individuals without clinical signs of atherosclerosis.

There are also studies showing the relationship between plasma salusin levels and atherosclerotic risk factors. In a study conducted on patients undergoing hemodialysis due to end-stage renal disease, it was found that a lipid-lowering diet, increase in physical activity, and atorvastatin treatment caused a decrease in LDL cholesterol and an increase in HDL/LDL ratio by increasing the level of salusin alpha in the plasma [14]. In a study involving type 2 diabetes patients, a negative correlation was found between plasma salusin alpha level and LDL cholesterol level [15]. Similar effects have been demonstrated in animal studies. Salusin alpha administered to mice with apolipoprotein E deficiency for eight weeks caused a serious decrease in total cholesterol serum level and an increase in HDL cholesterol level [7]. In another study, it was found that salusin alpha given in high doses for 12 weeks caused a decrease in total cholesterol, triglyceride, and LDL cholesterol levels and an increase in HDL cholesterol levels [12]. In both studies, it was observed that salusin alpha prevented the progression of atherosclerosis by causing an improvement in the serum lipid profile. It has been shown that the suppression of atherosclerosis results from the inhibition of foam cell formation from macrophages through the downregulation of the acetyl-coenzyme A acetyltransferase-1 (ACAT-1) enzyme [6]. The result of all these studies is that the increase in plasma salusin alpha level positively affects the lipid profile. In our study, in parallel with the previous literature, we found that participants with high plasma salusin alpha levels had low total cholesterol, triglyceride, and LDL and non-HDL levels. Non-HDL levels indicate all atherogenic lipoproteins with apo-B content in plasma [16]. The role of LDL cholesterol and other apo-B-containing lipoproteins in atherosclerotic processes is well known [17]. The absolute decrease in LDL cholesterol, independent of drugs, leads to a decrease in the risk of cardiovascular disease [18].

In our study, there was also a statistically significant correlation between smoking and serum salusin alpha levels. Salusin alpha levels were found to be significantly lower in smokers. We did not find any data on this relationship in the literature before. In the study by Nakayama et al. [19], it was found that Jak-2 (Janus kinases), which can be upregulated with stimuli such as angiotensin II, inflammatory cytokines, and atherogenic growth factors, suppresses the expression of salusin alpha. It has been emphasized that this mechanism is the possible reason for the decrease in serum salusin alpha levels in atherosclerosis. It has been previously shown that the Jak-2 V617F gene mutation is more pronounced and frequent in smokers compared to nonsmokers [20]. This mutation disrupts the autoinhibition of Jak-2, leading to the constitutive activation of the Jak-2 signaling pathway. The decrease in salusin levels seen in smoking participants may be due to the Jak-2 V617F gene mutation that occurs with smoking.

There are no studies evaluating the relationship between CVD risk and salusins in previously asymptomatic patients. To the best of our knowledge, our study is the first to evaluate plasma salusin alpha levels in asymptomatic individuals whose cardiovascular risk is determined using the SCORE2 and PCE algorithms. In our study, we found that SCORE2, PCE scores, and CV risk groups had a negative significant correlation with plasma salusin alpha levels. The relationship of this peptide, which has anti-atherogenic activity, with CV risk scores would contribute to the production of modified score systems that could be evaluated with clinical outcomes in the future. Salusin alpha is associated not only with CV risk scores but also with participants’ scores for deviation from baseline risk status (Δ SCORE2 and Δ PCE) based on age, sex, and ethnicity. This negative correlation has a stronger correlation than standard scores. This result can be interpreted that salusin alpha can also be used in the follow-up of CV risk reduction with lifestyle changes or in determining the severity of the calculated CV risk.

Current guidelines do not recommend the use of any biochemical parameter other than non-HDL cholesterol for use in the SCORE2 and PCE algorithm [2,3]. There are previous studies conducted on patients with some biochemical parameters and SCORE risk groups. In a study including non-diabetic asymptomatic patients, it was found that HbA1c indicates the prevalence of subclinical atherosclerosis in patients in the low-intermediate risk group according to the SCORE index [21]. In another study, it was shown that serum nitric oxide (NO) level has a significant negative correlation with systolic and diastolic blood pressure in asymptomatic patients who are in the low cardiovascular risk group according to the SCORE score. It was emphasized that the increase in serum NO in this group indicates a decrease in blood pressure. In the study, while NO levels did not show a significant difference in the group with and without CAD, endothelin levels were found to be significantly higher in men with CAD than in those without CAD [22]. In another study involving liver transplant candidates, asymmetric dimethylarginine (ADMA) and NO levels were classified according to the SCORE index, and it was shown that they were significantly higher in the group with high CV risk. In the study, it was emphasized that the increase in ADMA and NO levels was independently associated with the increase in CV risk [23]. The authors, who integrated the PCE CV risk scoring system with isolated post-challenge hyperglycemia in postmenopausal women, created the opportunity to perform a more sensitive risk analysis on the participants with the modified assessment system they obtained [24]. Our study provides valuable information to express the diagnostic effects of biomarkers in CV risk classification. The decrease in salusin alpha plasma levels appears to lead to an increased risk of CVD. The fact that exogenous salusin alpha suppressed atherosclerosis in animal experiments also shows how important our findings are. In the coming years, it is clear that more comprehensive studies are needed to comment on whether salusin alpha can be an effective biochemical marker in CV risk assessment processes and whether exogenous administration, among other risk measures, will reduce the risk in patients with a high risk of CV event development.

Limitations

The most obvious limitation is that the study was single-centered with single ethnicity, and the number of participants was relatively low. In addition, our study was carried out in an ethnicity considered a high-risk group in the SCORE2 algorithm. Whether there is underlying subclinical atherosclerosis in the patient population has not been revealed by imaging methods. The study includes hypertensive patients, and some of them use antihypertensive drugs. The cardiovascular risk level is lower in patients whose blood pressure is under control. It is not clear whether antihypertensive drugs have a direct effect on salusin alpha levels in the low CV risk group.

## Conclusions

In conclusion, in our study, we found that salusin alpha has a negative correlation with SCORE2 and PCE risk scores. It has significant diagnostic power in the prediction of CV risk groups. Salusin alpha is an independent variable that has a risk-reducing effect in the distribution of risk groups. It was also found that salusin alpha had a negative and significant relationship with the severity and prevalence of CV risk factors.
